# A Method for Paired Comparisons of Glo Germ Quantity in Images of Hands Before and After Washing

**DOI:** 10.3390/jimaging12040178

**Published:** 2026-04-21

**Authors:** Jordan Ali Rashid, Stuart Criley

**Affiliations:** 1 Department of Cognitive Sciences, UC Irvine, Irvine, CA 92697, USA; 2 Indelible Learning, Rolling Hills Estates, CA 90274, USA

**Keywords:** Glo Germ™, fluorescence detection, color imaging

## Abstract

We present a reproducible pipeline that converts color images into quantitative fluorescence maps by combining spectral measurement with a linear mixture model. The method is designed specifically for quantitative comparisons of Glo Germ™ on images of hands taken under different experimental conditions with controlled illumination. The emission spectrum of Glo Germ is measured using a spectral photometer and normalized to obtain its spectral power density function. This spectrum is projected into CIE XYZ coordinates and incorporated into a linear mixture model in which each pixel contains contributions from white light, UV-illuminated skin reflectance, and fluorophore emission. Component magnitudes are estimated with non-negative least squares, yielding a grayscale image whose intensity is a monotonic proxy for local fluorophore density. Spatial integration provides an image-level summary proportional to total detected material. Compared with single-channel proxies, the observer suppresses background structure, improves contrast, and remains radiometrically interpretable. Because the method depends only on measurable spectra and linear transforms, it can be reproduced across cameras and extended to other fluorophores.

## 1. Introduction

Fluorescent tracers such as Glo Germ™ (Glo Germ, Moab, UT), which uses 5-μm melamine copolymer resin beads suspended in hand lotion, are widely used to visualize contamination and transfer in hygiene training, biosecurity exercises, and clinical education [1,2,3,4,5,6,7,8]. Under near-ultraviolet illumination, the material emits a distinctive blue fluorescence that allows instructors and researchers to visually demonstrate how contaminants spread across surfaces and hands. In many of these applications, the central question is not simply whether fluorescence is present, but whether hygiene interventions—such as hand washing—reduce the amount of contaminant present on the skin. The practical task is therefore a paired comparison problem: the same hands are photographed before and after washing, and the effectiveness of the intervention is evaluated by comparing the amount of fluorescent material visible in the images.

Despite the widespread use of Glo Germ in such demonstrations, quantitative analysis of the resulting images remains largely heuristic. Many studies rely on visual inspection or simple image-processing proxies such as luminance measurements or single-channel color thresholds [9]. These approaches can provide rough indicators of contamination but do not explicitly account for the physics of fluorescence imaging. In particular, the intensity recorded by a camera sensor may include contributions not only from fluorescent emission but also from reflected illumination, skin reflectance, and other environmental light sources. As a result, simple image metrics can conflate fluorescence with background illumination, making it difficult to obtain consistent quantitative measurements.

Deochand et al. [10,11] used image-based fluorescence quantification based on a luminance proxy. They were motivated to find a method that did not rely on trained observers to rate the effectiveness of hand-washing. Their solution was to use the luminance tool based in Adobe Photoshop™ and apply it to each photo individually. Therefore, their solution not only requires access to expensive software; it applies a unique transformation to each image. Furthermore, as we will explore later, luminance (indeed, any single-channel proxy obtained by linear transformation) may include other sources of light besides fluorescence. Even under controlled conditions, these alternative light sources must be accounted for, and where possible, their effects should be removed from the final result.

At the same time, a well-established body of work in signal detection theory has shown that when the spectral signature of a target is known, and the measurement noise is approximately additive and Gaussian, the optimal linear detector is a matched filter, producing a scalar sufficient statistic that maximizes signal-to-noise ratio [12]. Closely related formulations appear in multispectral and hyperspectral imaging, where spectral unmixing and matched filtering are routinely used to separate known material signatures from background clutter [13,14,15,16].

In the present study, we focus on a more restricted but practically important problem: quantifying the difference in fluorescent material on hands before and after washing under controlled illumination conditions. Our goal is not to develop a general-purpose fluorescence detector for arbitrary scenes, but rather to provide a reproducible and physically interpretable measurement of Glo Germ density under a fixed imaging setup with our illuminant. Under these conditions, the same illumination, camera, and geometry are used for case-matched images, allowing the change in fluorescence signal to serve as a quantitative indicator of the effectiveness of the washing procedure.

From a physical standpoint, fluorescence emission under controlled excitation conditions is additive and spectrally stable. As the density of fluorophore on a surface increases, the emitted radiance increases while the spectral shape of the emission remains approximately constant. When fluorescence occurs at the surface rather than within a volumetric medium, the observed light can be modeled as a linear combination of reflected illumination and fluorescent emission over a broad range of surface densities. This additive structure makes it possible to treat the measured color signal as a mixture of underlying spectral components.

In this work, we use this observation to construct a spectrally informed model observer for estimating the fluorescent component of camera images. Here, a model observer refers to a computational estimator that maps image measurements to a scalar detection or estimation statistic based on an assumed image formation model. The emission spectrum of Glo Germ is measured directly using a spectral photometer and normalized to obtain its spectral power density function (SPDF). We then project this spectrum into CIE XYZ color space and construct a linear mixture model in which each pixel is assumed to contain contributions from three physically motivated sources: broadband white light, ultraviolet-illuminated skin reflectance, and fluorescent emission. The relative magnitudes of these components are estimated using non-negative least squares. The resulting fluorescence coefficient provides a spatial map of fluorophore contribution across the image, and spatial integration of this coefficient yields a scalar value proportional to the total detected fluorescence.

Within the paired comparison framework considered here, this scalar measurement provides a rigorous quantitative metric for evaluating changes in fluorescent contamination between images of the same subject taken under identical imaging conditions. By grounding the detector in measured spectra and a linear radiometric model, the proposed approach replaces heuristic image processing with a reproducible and physically interpretable measurement pipeline. The method is designed specifically for controlled experimental settings such as hygiene training studies, where the goal is to compare contamination levels before and after an intervention rather than to detect fluorescence in arbitrary scenes.

### 1.1. Contributions

The contributions of this study are intentionally focused on the quantitative analysis of Glo Germ fluorescence under controlled imaging conditions rather than the development of a general-purpose fluorescence detection system. Specifically, this work makes the following contributions:

1. **A spectrally informed measurement framework for Glo-Germ fluorescence**. We measure the emission spectrum of Glo Germ under ultraviolet excitation and represent it as a normalized spectral power density function. This measured spectrum is then projected into CIE XYZ color space, allowing the fluorescent signal to be incorporated into a physically interpretable colorimetric model.
2. **A linear mixture model for separating fluorescence from background reflectance caused by human skin**. The observed camera signal is modeled as a linear combination of three components: broadband white light, ultraviolet-illuminated skin reflectance, and fluorophore emission. Estimating the coefficients of this model using non-negative least squares yields spatial maps that partition the measured light into physically meaningful sources.
3. **A quantitative fluorescence metric for paired comparisons of hygiene interventions**. The estimated fluorescence coefficient provides a grayscale image representing the spatial distribution of Glo-Germ emission. Spatial integration of this image yields a scalar value proportional to the total fluorescent material in the field of view. Under controlled illumination and imaging geometry, this metric enables rigorous quantitative comparison of Glo-Germ levels on hands before and after washing.
4. **A reproducible pipeline compatible with commodity cameras**. By operating in CIE XYZ space and using measured spectral signatures, the proposed method can be reproduced across camera systems capable of producing linearized RAW data, making it suitable for controlled experimental studies of hygiene effectiveness.

Taken together, these contributions demonstrate how spectral measurement and linear color modeling can be combined to transform fluorescence photographs into reproducible quantitative measurements for controlled paired-comparison experiments.

### 1.2. Main Differences with Existing Literature

#### Linear Range

In applications of fluorescent imaging, the goal is to detect the presence or concentration of a substance in images of biological samples. Macroscopic applications are usually medical and require specialized detection equipment and highly trained technologists to operate it. In microscopic applications, models use low-concentration samples of the substance to estimate the concentration in a range where it is linear with light emission. At higher concentrations, there is an inner filter effect that follows the Beer–Lambert law. This effect occurs because fluorophores are also chromophores, causing the substance to reabsorb some of its own light emissions.

In the present application, we are only interested in the light radiating from the surface of the sample, not the absorption of light as it passes through the sample, so inner-filtering is not a concern at high concentrations. In other words, we are interested in the emission of light at the macroscopic level, not the transmission of light at the microscopic level. In our setup, linearity is achieved at high densities of Glo Germ where the chromaticity of the observed light remains constant and the intensity monotonically increases with fluorophore density.

The deviation from linearity that we do need to worry about occurs at low concentrations of Glo Germ, where it is spread so thinly over human skin that the reflectance spectrum of human skin begins to contribute to the radiated light spectrum. Fortunately, the fluorescent emissions combine additively with this background noise, and thus the effect can be removed by subtraction:

radiance=(reflectance×illuminant)+fluorescence

## 2. Model

In terms of the spectral radiance of the illuminated surface (i.e., the stimulus),

(1) I(λ)=S(λ)+F(λ)

(2) $$=\alpha_{s}s\left(\lambda \right)+\alpha_{f}f\left(\lambda \right)$$

where F(λ) is the Glo Germ emission spectrum, S(λ) is the component of light visibly reflecting off the surface of the skin, s(λ) is the spectral power density function (SPDF) of the surface without any Glo Germ, and f(λ) is the SPDF of the fluorescent emission spectrum. Formally, we can express the relationships between these quantities in the following way:

$$\alpha_{f}=\int_{V}F\left(\lambda \right)d\lambda \;\;\;\mathrm{and}\;\;\;f\left(\lambda \right)=\frac{F(\lambda )}{\alpha_{f}}$$

$$\alpha_{s}=\int_{V}S\left(\lambda \right)d\lambda \;\;\;\mathrm{and}\;\;\;s\left(\lambda \right)=\frac{S(\lambda )}{\alpha_{s}}$$

where the domain of integration *V* represents the visible spectrum (390 to 830 nanometers in our calculations). This was the widest possible range we could use due to the tabulated values of our color-matching functions being limited to this range. In addition, it is unlikely that extending the range would change the results because the color-matching functions are zero outside this range.

Division by α is equivalent to normalization, so the new integral of the function over the visible spectrum is equal to 1. This is called the spectral power density function (or SPDF) [17]. The spectral power density functions *s* and *f* can be thought of as conditional probability distributions over radiated photon wavelengths given the source. In other words, if one were to grab a random photon from the light radiating from a source, what is the probability (density) of it having a particular wavelength? The answer is the spectral power density function. By definition, this means that α is the (visible) radiant power of the source. Essentially, we are modeling light spectra I(λ) as histograms generated from linear mixtures of source SPDFs. For example, $\alpha_{f}$ represents the total power radiating from the stimulus due to fluorescence, and f(λ) tells us how much of that energy is attributed to photons with wavelength λ.

Consideration of the mechanics will show that the parameter $\left(\alpha_{s},\alpha_{f}\right)$ depends on the density of Glo Germ on the surface. When density is zero, $\alpha_{f}=0$ and $\alpha_{s}$ is the radiant power of the stimulus. In this case, the visible light is entirely due to the illuminant reflecting off the naked surface. As Glo Germ density increases, $\alpha_{f}$ increases as more of the radiant light is attributed to the fluorescence. Immediately, $\alpha_{s}$ begins to decrease as the Glo Germ absorbs more of the available light before it can irradiate the surface. At a critical density, when Glo Germ absorbs all of the illuminant, $\alpha_{s}=0$, and $\alpha_{f}$ is the radiant power of the stimulus. In this case, the visible light is entirely due to the fluorescence of Glo Germ. Increasing the amount of Glo Germ will yield diminishing returns on the radiant power. Previous research indicates that the critical density and asymptotic power both depend on the surface substance [3].

Equation (1) applies to spectra; thus, if *L* is a linear filter applied to the spectral radiance I(λ), then

(3) $$L\left(I\right)=\alpha_{s}L\left(s\right)+\alpha_{f}L\left(f\right)$$

**Figure 1:**
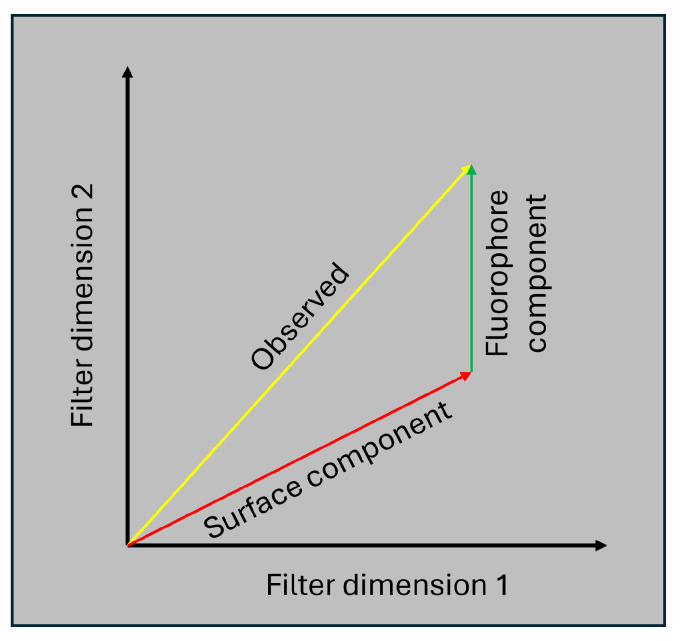
A 2D representation of the decomposition based on vector additivity of lights. The yellow arrow represents the vector of total light observed by the camera sensor. The red arrow represents the vector of light contributed by the illuminant reflected off the naked surface. The green arrow represents the vector of light contributed by the Glo Germ emissions.

This means that if L(s), L(f), and L(I) are known, then we can estimate α by projecting L(I) onto the basis spanned by [L(s),L(f)]. If we assume normally distributed errors, then this becomes a linear regression problem. Figure 1 shows a graphical depiction of this idea in a 2-dimensional color space.

### Goal and Outputs

The model observer would have a single spectral sensitivity function optimized for the detection of the fluorescent color in raw camera files. Operationally, it:

1. programmatically “observes” every pixel of an XYZ colorspace image projected onto a sensor;
2. converts it to a grayscale detection image where brightness is a direct indicator of Glo Germ density;
3. reduces each image to a scalar proportional to the integral of Glo Germ density over the captured area.

## 3. Materials and Methods

### 3.1. Equipment

The ultraviolet light source was constructed with an array of 165 0.5 Watt gallium nitride [18] SMD2835 LEDs on adhesive strips, emitting UVA light over a narrow band of wavelengths from 395–405 nm (YGS-Tech, Hong Kong). This array was mounted in 2 banks of LEDs in the same plane and on either side of the camera in an enclosed box, with a narrow slot at the bottom that would allow a subject’s hands and fluorescent Glo Germ to be imaged. Both the camera and the LEDs were approximately 30 cm from the subject. The camera used to photograph Glo Germ and subjects’ hands was a consumer-grade Sony ZV-1 (Sony Corporation, Tokyo, Japan) with a 1-inch Exmor RS stacked CMOS image sensor, set to manual ISO speed, aperture, and exposure that outputs raw files in 12-bit RGB color.

We have included an Excel™ file in the Supplementary Materials (GlowSpectra.xlsx) with the measured illuminant sampled over the visible spectrum. In addition, we have included a MATLAB™ R2024b function (TargetFilter.m), which takes no input arguments and returns the measured illuminant as the final output argument.

### 3.2. Spectral Power Density Function of the Glo Germ Emission Spectrum

**Figure 2:**
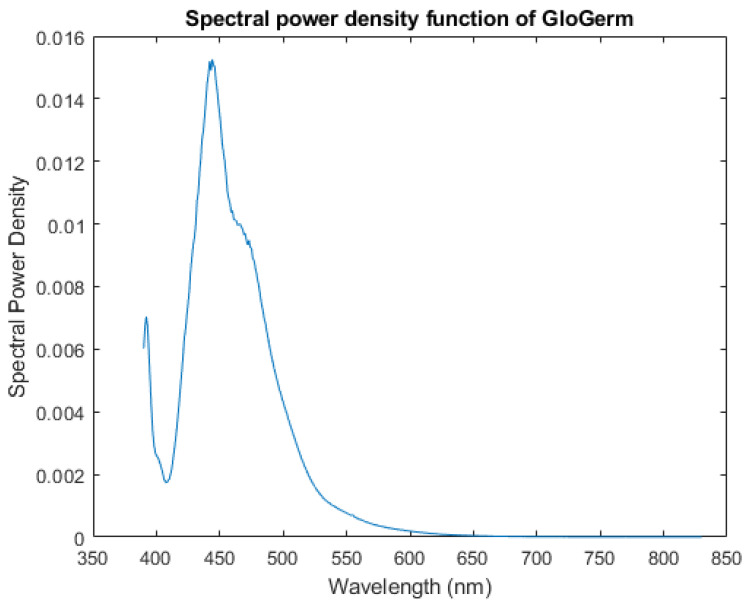
The Glo Germ fluorescent emission spectrum normalized to integrate to 1 (i.e., spectral power density function). The SPDF determines the optimal combination of any filter basis to represent the color of the Glo Germ excited by the UV lamp. It has been sampled linearly from 390 to 830 nm. Notice the second peak below 400 is not due to fluorescence per se, but rather reflection. Regardless, we consider it part of the visible action.

Fluorescence emission is additive. As the amount of Glo Germ increases, the emission spectrum will retain the same shape. Thus, we are mainly interested in the shape (chromaticity) of the Glo Germ emission spectrum. For this reason, we renormalize the measured fluorescent emission spectrum to integrate to 1. This preserves the shape of the spectrum. It is called the spectral power density function, or SPDF, shown in Figure 2.

(4) $$\hat{f}\left(\lambda \right)=\frac{\hat{F}\left(\lambda \right)}{\int_{V}\hat{F}\left(x\right)dx}$$

where the domain of integration *V* is the visible spectrum (390 to 830 nanometers in our calculations), and $\hat{F}$ is the measured Glo Germ emission spectrum (see Appendix A for details).

We have included a MATLAB™ function in the Supplementary Materials (TargetFilter.m) which returns the Glo Germ SPDF as the first output and the measured Glo Germ emission spectrum as the third output. The reader can also find this measured spectrum in the Excel™ file GlowSpectra.xlsx.

### 3.3. Photo Test Strip of Glo Germ

To characterize the response of the fluorescent Glo Germ under our UV LED setup, we applied 0.3 mL swatches of Glo Germ diluted in glycerin onto black felt. The concentration of Glo Germ in each swatch ranged from 100% to 0%. Each dilution was 15/65ths of the previous sample, or approximately 23.07%, creating a set of samples that spanned 7 logs of concentration. The resulting panel of Glo Germ swatches was photographed with the UV LED and camera setup at ISO 1000, f/2, with exposures ranging from 2 s down to 1/50th of a second. We have included a folder in the Supplementary Materials (“Response Curve Photos”) which contains the test strip photos at each exposure duration.

We screened many potential substrates before using 100% polyester black felt. Fabric that has been laundered will be contaminated with optical brighteners [19] with fluorescence that rivals that of Glo Germ. Even black paper was found to be contaminated with flecks of fluorescent fiber. For these reasons, when photographing hands with or without Glo Germ, care must be taken to reduce extraneous sources of fluorescence, including clothing, bracelets, and rings.

### 3.4. SPDF of Human Skin Under Illuminant

The U.S. National Institute of Standards and Technology [20] has spectral reflectance data from 100 human subjects available here: https://www.nist.gov/publications/reference-data-set-human-skin-reflectance (accessed on 2 April 2026). We can use these data along with measurements of our UV illuminant to determine the radiance of human skin illuminated by our UV lamp.

**Figure 3:**
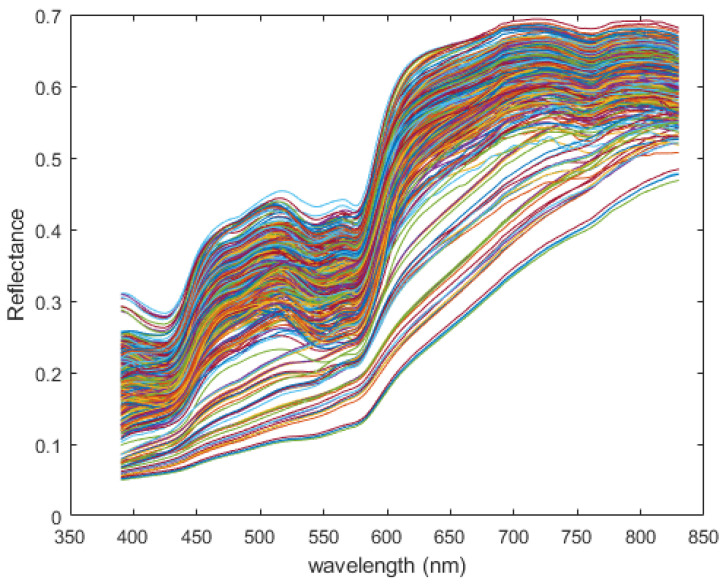
Spectral reflectance of 100 human subjects (plotted in different colors), made available by NIST. Values are shown over the visible spectrum only.

**Figure 4:**
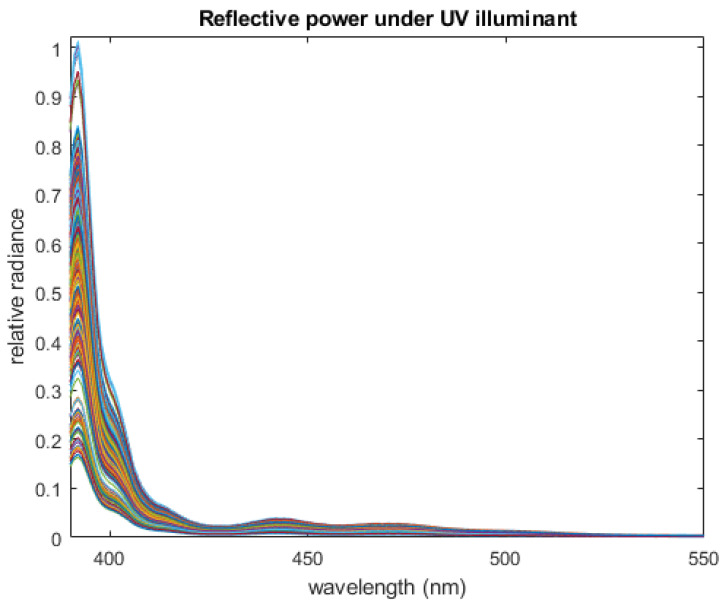
Spectral radiance of NIST subjects’ skin illuminated by our UV lamp. Each color is for a different subject.

**Figure 5:**
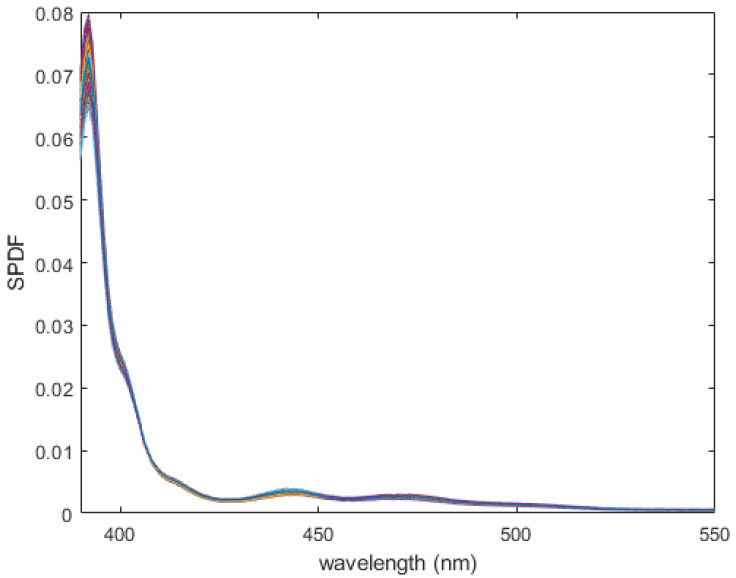
Spectral power density functions for the 100 NIST subjects. Each curve is normalized to integrate to 1, thus containing only the chromatic direction of lights that can be produced from the UV lamp radiating off the NIST subjects’ skin. Each color is for a different subject.

Let $R_{k}\left(\lambda \right)$ be the spectral reflectance of subject *k* (these curves are shown in Figure 3), and let P(λ) be the spectral radiance of our UV lamp. Then the spectral radiance of the skin of subject *k* illuminated by our lamp will be $S_{k}\left(\lambda \right)=R_{k}\left(\lambda \right)P\left(\lambda \right)$ (these curves are shown in Figure 4). Notice that these curves are largely similar in shape, and appear to differ mostly in scale, especially at wavelengths less than 400 nm. This variation is largely squashed when the curves are re-normalized to produce spectral power density functions for each subject, $s_{k}\left(\lambda \right)$ (shown in Figure 5).

Compared to the differences in the spectral reflectance and radiance of human skin shown in Figure 3 and Figure 4, there is almost no variation in the individual SPDFs shown in Figure 5. This is due simply to the fact that the UV lamp is narrowband. Since there is not a lot of variation in the photons irradiating the skin surface, there will not be a lot of variation in the chromaticity of illuminated skin. Hence, we can take the mean of the individual SPDFs to obtain the standard:

(5) $$\hat{s}\left(\lambda \right)=\frac{1}{100}\sum_{k=1}^{100}s_{k}\left(\lambda \right)$$

where

$$s_{k}\left(\lambda \right)=\frac{R_{k}\left(\lambda \right)P\left(\lambda \right)}{\int_{V}R_{k}\left(x\right)P\left(x\right)dx}$$

We have included a MATLAB™ function in the Supplementary Materials (TargetFilter.m) which returns the SPDF of human skin as the second output.

The radiance of human skin illuminated by our UV lamp comprises mostly ultraviolet light less than 400 nm. Inspection of the SPDF for Glo Germ shown in Figure 2 will show a similar peak at ultraviolet wavelengths. In other words, some (but not all) of the observed light formed from mixtures of skin and Glo Germ will not be separable—meaning the inner products of *f* and *s* with the light *I* will be correlated. However, given the amount of energy radiating from the Glo Germ at wavelengths greater than 400 nm, it is still possible to filter the two sources.

### 3.5. From SPDFs to Tristimulus Values

#### 3.5.1. Camera-Oriented Basis (CIE XYZ as a Camera Proxy)

Our camera pipeline represents color in or near a CIE XYZ-like color space basis after demosaicing, white balance, and color correction, using the measurements taken from the camera at the time of recording. When working with RAW files, it is generally preferable to remain in a linear sensor space (raw RGB after black-level correction and normalization) and treat XYZ as a reference basis for interpreting the target color, rather than as the computational basis for detection. For reproducibility across other types of camera, we implement detection in XYZ color space. Our camera has the following matrix transformation from linear RGB values to CIE XYZ:

(6) $$\left[\begin{array}{c} X\\ Y\\ Z \end{array}\right]=\left[\begin{array}{ccc} 1.3565 & 0.3424 & 0.0420\\ 0.4073 & 1.0098 & -0.3516\\ 0.0222 & -0.2577 & 1.9889 \end{array}\right]\left[\begin{array}{c} R\\ G\\ B \end{array}\right]$$

The matrix in Equation (6) was obtained from the camera’s DNG color calibration metadata. We have included a MATLAB™ function in the Supplementary Materials (raw2xyz.m) which takes a RAW file name as input and returns the image in XYZ coordinates (in addition to the original RGB image and the color transformation matrix). With this function, the reader may apply our method to a series of RAW files. The third output argument would replace the matrix in Equation (6).

#### 3.5.2. Spectral Function to Color Coordinates

**Figure 6:**
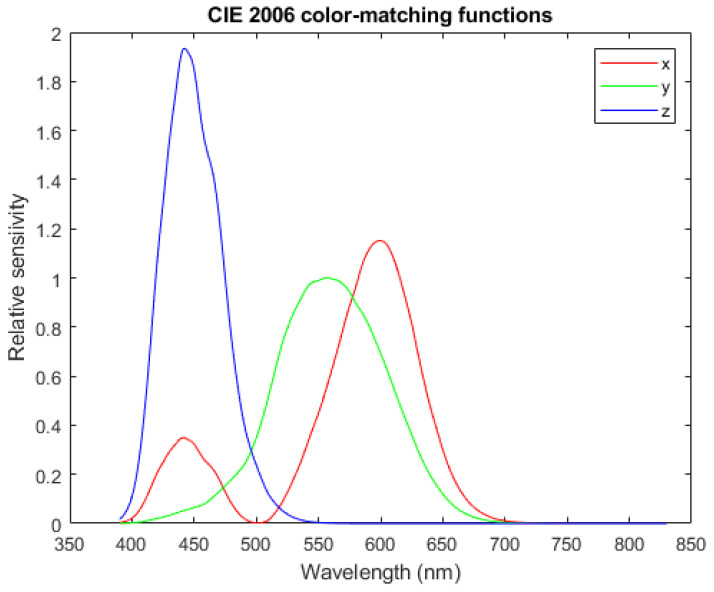
$\bar{x}$, $\bar{y}$, and $\bar{z}$ color-matching functions used to determine the chromaticity of the Glo Germ emission spectrum.

It is possible to calculate tristimulus values identifying the color direction of lights produced by linear combinations of the SPDFs under any set of color-matching functions. For example, using the CIE 2006 $2^{\circ }$ XYZ color matching functions $\left\{\bar{x}\left(\lambda \right),\bar{y}\left(\lambda \right),\bar{z}\left(\lambda \right)\right\}$ (shown in Figure 6),

(7) $$\begin{array}{c} L\left(t\right)=\int_{V}\left[\begin{array}{c} \bar{x}\left(\lambda \right)\\ \bar{y}\left(\lambda \right)\\ \bar{z}\left(\lambda \right) \end{array}\right]t\left(\lambda \right)d\lambda \end{array}$$

Our design matrix is:

(8) $$\left[L\left(w\right),L\left(\hat{s}\right),L\left(\hat{f}\right)\right]=\left[\begin{array}{ccc} 1.000 & 0.0914 & 0.2149\\ 1.000 & 0.0702 & 0.1614\\ 1.000 & 0.3381 & 1.1198 \end{array}\right]$$

These coordinates are universal—meaning they apply to any camera’s output, properly transformed to XYZ coordinates. We have included a MATLAB™ function in the Supplementary Materials (LoadCameraFilters.m) which requires no input arguments and returns a structure containing the sampled wavelengths and the values of the XYZ color-matching functions at each wavelength. With the fields of this structure, the reader can map spectral data directly to XYZ tristimulus values.

### 3.6. A Matched Filter Response Model for Estimating Fluorescence

The matched filter response is the optimal linear filter in the sense that it maximizes the signal-to-noise ratio under Gaussian noise assumptions. It is equal to the inner product of $L(\hat{f})$ and L(I):

(9) $$g(I)=L{\left(\hat{f}\right)}^{T}L\left(I\right)$$

(10) =0.2149X+0.1614Y+1.1198Z

where the coefficients in the second line are specific to the XYZ color-matching functions we used.

The matched-filter response is the simplest path to a single-channel linear proxy for Glo Germ concentration, but it is problematic because it confuses other sources of light and noise as Glo Germ. In this way, it is equivalent to any other single-channel response such as luminance. However, it is theoretically optimal in the sense that the direction of the axis upon which we project colors is based on the SPDF of Glo Germ.

We have included a MATLAB™ function in the Supplementary Materials (LoadDecompositionMatrix.m) that returns these coefficients as the second output.

### 3.7. A Model for Estimating Fluorescence Separated from Other Light Sources

The matched filter response model is the simplest model for our purpose, but it suffers from limitations due to confusion with alternative light sources. In this section, we introduce an alternative that seeks to separate the contribution of Glo Germ from white light and skin reflectance. Without loss of generality, we can rewrite Equation (3) as

(11) $$\begin{array}{cc} L(I) & =\beta_{w}L\left(w\right)+\beta_{s}L\left(\hat{s}\right)+\beta_{f}L\left(\hat{f}\right)+\varepsilon \end{array}$$

$\beta_{w}$ represents the contribution of white light, and the vector ε represents the contribution of (1) other systematic sources of light not in the subspace spanned by the basis and (2) random noise, which we assume is based on large numbers of photons behaving according to photon noise (i.e., approximately normal).

When the solution to Equation (11) is unconstrained, the estimated regression coefficient $\beta_{f}$ is determined by the inner products $L{\left(w\right)}^{T}L\left(I\right)$, $L{\left(\hat{s}\right)}^{T}L\left(I\right)$, and $L{\left(\hat{f}\right)}^{T}L\left(I\right)$. More specifically, $\beta_{f}$ is linearly increasing with the matched-filter response, $L{\left(\hat{f}\right)}^{T}L\left(I\right)$. The exact linear relationship is moderated by terms $L{\left(w\right)}^{T}L\left(I\right)$ and $L{\left(\hat{s}\right)}^{T}L\left(I\right)$.

Let c(x,y) be the XYZ tristimulus value measured by the camera sensor at location (x,y), and let $\beta \left(x,y\right)\in R_{+}^{3}$ represent the projection components onto the subspace spanned by the basis in Equation (8).

(12) $$c\left(x,y\right)=\left[\begin{array}{ccc} 1.000 & 0.0914 & 0.2149\\ 1.000 & 0.0702 & 0.1614\\ 1.000 & 0.3381 & 1.1198 \end{array}\right]\;\beta \left(x,y\right)+\left[\begin{array}{c} \varepsilon_{X}\\ \varepsilon_{Y}\\ \varepsilon_{Z} \end{array}\right]$$

We use non-negative least squares [21] to partition the light measured by pixel location (x,y) into (1) white light, (2) light reflected by the surface of the skin, and (3) light emitted by fluorophores.

We have included a MATLAB™ function in the Supplementary Materials (LoadDecompositionMatrix.m) that returns this array as the first output. In addition, we have included a MATLAB function (glowfun_decomp.m) which takes an XYZ image and the 3-by-3 array as inputs, and returns the coefficients $\beta_{w}$, $\beta_{s}$, and $\beta_{f}$ as the first, second, and third outputs. The fourth output is the vector of residuals.

## 4. Results

### 4.1. Response Curves and Exposure Duration

**Figure 7:**
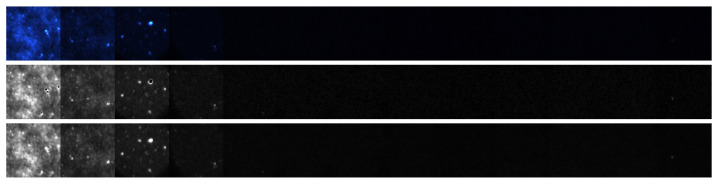
Images of the test strip obtained from a 0.02 s exposure duration. The **top** row shows the RGB color image. The **middle** row shows the non-negative least squares estimate. The **bottom** row shows the matched filter response.

**Figure 8:**
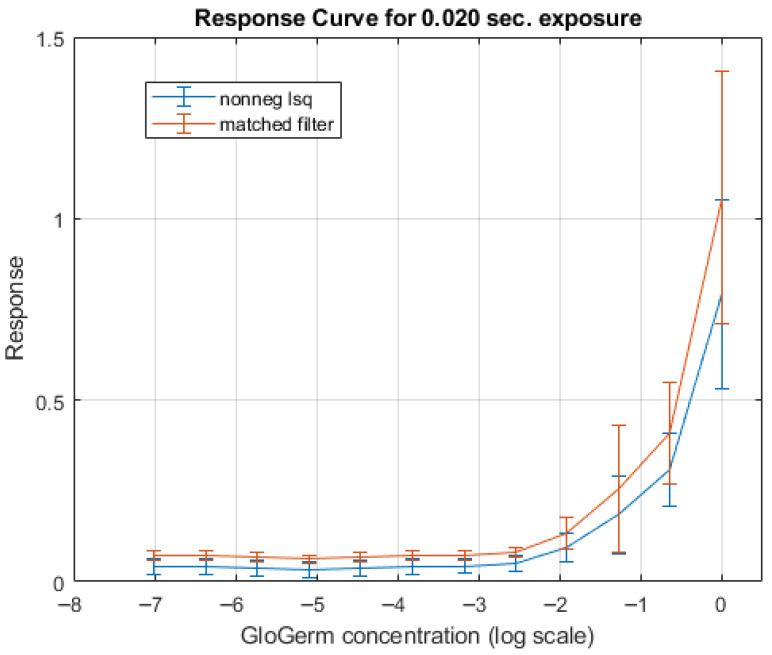
Response curve generated from the test strip photo shown in Figure 7. Notice the responses do not begin to rise until roughly 1% concentration.

**Figure 9:**
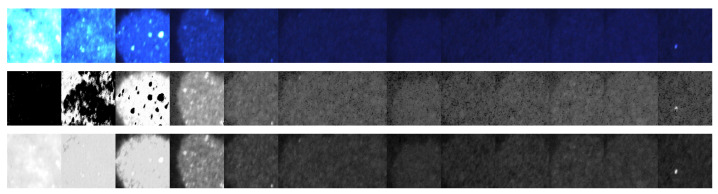
Images of the test strip obtained from a 0.2 s exposure duration. The **top** row shows the RGB color image. The **middle** row shows the non-negative least squares estimate. The **bottom** row shows the matched filter response.

**Figure 10:**
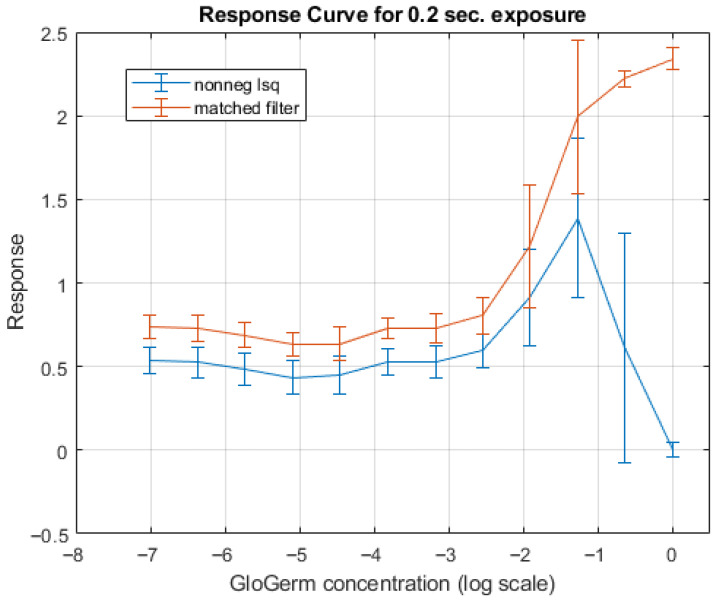
Response curve generated from the test strip photo shown in Figure 9. Notice the catastrophic failure of the non-negative least squares estimator, and the saturation of the matched filter response.

The response of both the matched filter statistic and the non-negative least squares (NNLS) estimator depends strongly on the exposure conditions under which the image is acquired. Figure 7, Figure 8, Figure 9 and Figure 10 illustrate how the estimated fluorescence signal varies as a function of the underlying fluorophore contribution under different exposure regimes.

**Figure 11:**
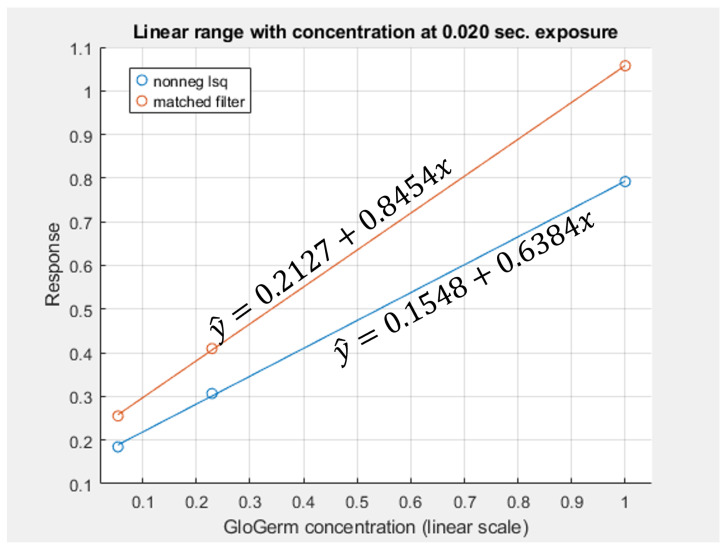
Response curves for the 0.02 s exposure, shown in linear scale over the range where response is approximately linear with concentration. For the non-negative least squares estimate, the $R^{2}$ for the shown regression line is 0.9997. For the matched filter, the $R^{2}$ is 0.9999.

At low exposure levels, both estimators exhibit approximately monotonic response curves with respect to increasing fluorescence. In this regime, the measured tristimulus vector remains within the linear operating range of the camera sensor, and the assumptions of the linear mixture model are satisfied. As a result, both the matched filter response and the NNLS estimator provide meaningful discrimination between different fluorescence levels. It appears either estimator can discriminate between concentrations greater than 1%. Furthermore, as can be seen in Figure 11, the responses are roughly linear with concentrations above 5%.

As exposure increases, the behavior of the two estimators diverges. The matched filter response exhibits a gradual saturation effect, in which increases in fluorescence produce diminishing changes in the output statistic. This behavior arises because of saturation of the camera sensors. Importantly, this saturation does not produce a catastrophic failure; rather, it limits the dynamic range over which fluorescence levels can be distinguished.

In contrast, the NNLS estimator is more sensitive to violations of the linear radiometric model that occur at high exposure levels. As the sensor approaches saturation, the measured color shifts toward the white-light direction, and the decomposition increasingly attributes signal to the broadband component rather than fluorescence. This results in a breakdown of the estimator, as seen in the response curves in Figure 9, where the estimated fluorescence can decrease or become unstable at high intensities.

### 4.2. Mean-Squared Error of Estimators for $\alpha_{f}$

We used the model of radiance in the second line of Equation (1) to simulate light spectra in the basis spanned by skin reflectance and Glo Germ fluorescence. In the first simulation, we used a fixed value of $\alpha_{s}=1$ and varied $\alpha_{f}$ between 0 and 10. In the second simulation, the total radiance was fixed ($\alpha_{s}+\alpha_{f}=10$). In both simulations, we passed the simulated spectrum through our color-matching functions and added Gaussian noise with a variable noise magnitude σ (note that the units on this noise magnitude match the units of the color-matching functions). The goal is to compare the outputs of each model in terms of their ability to recover the true physical fluorescent power, $\alpha_{f}$.

In both simulations, the error histograms are different across estimator types. For the non-negative least squares estimator, error histograms are bimodal. The first mode occurs whenever $\beta_{f}=0$, and the second mode occurs whenever $\beta_{f}\approx \alpha_{f}$. For the matched filter response, error histograms appear normally distributed (this outcome is expected from the matched filter in the presence of normal noise). The mean of the matched filter error histogram is never zero because it consistently overestimates the true value of $\alpha_{f}$.

**Figure 12:**
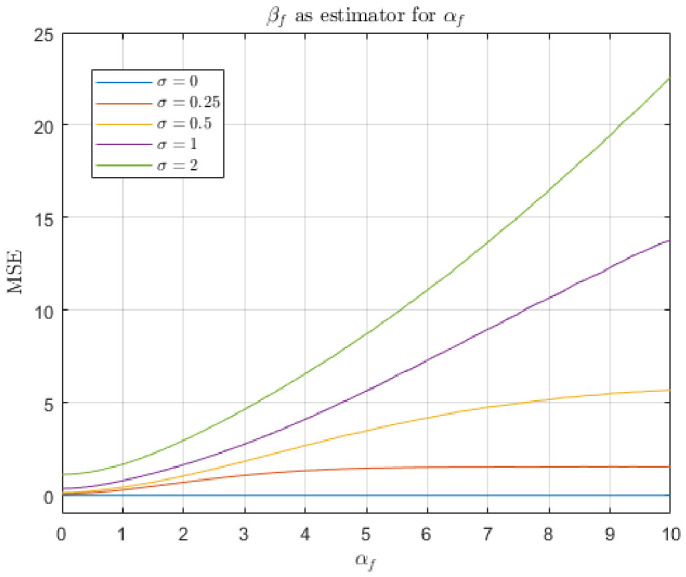
Mean-squared error of the non-negative least squares estimator for $\alpha_{f}$, collected while varying $\alpha_{f}$ and the additive noise magnitude σ. The skin radiance $\alpha_{s}$ was fixed at 1.

**Figure 13:**
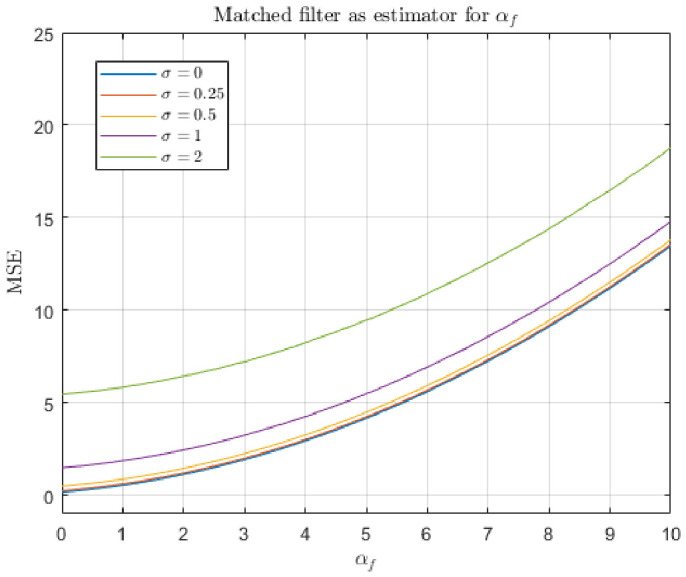
Mean-squared error of the matched filter response estimator for $\alpha_{f}$, collected while varying $\alpha_{f}$ and the additive noise magnitude σ. The skin radiance $\alpha_{s}$ was fixed at 1.

The results of the first simulation are shown in Figure 12 and Figure 13. In this scenario, the total radiant power of the input light is free to vary. We can see that for both estimators, MSE tends to increase with $\alpha_{f}$, which corroborates our previous result that lower incident intensities are preferable. However, the two estimators differ in the details. Figure 12 shows that the non-negative least squares estimator perfectly recovers $\alpha_{f}$ when there is zero noise. It outperforms the matched filter response in low-noise regimes (i.e., σ<1). In these regimes, MSE appears to be asymptotic with increasing $\alpha_{f}$. Such an asymptote does not occur with the matched filter response model. The matched filter response model performs about the same for all low-noise levels. In the zero-noise condition, the error is caused by confusion between skin reflectance and Glo Germ fluorescence.

**Figure 14:**
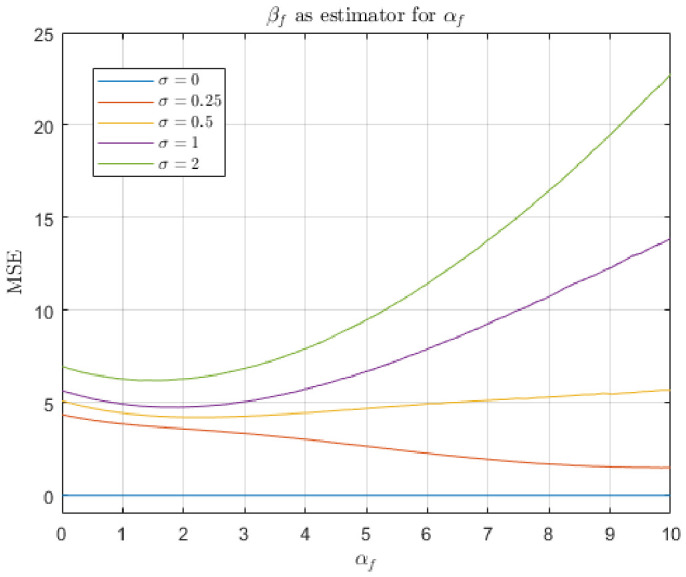
Mean-squared error of the non-negative least squares estimator for $\alpha_{f}$, collected while varying $\alpha_{f}$ and the additive noise magnitude σ. The skin radiance $\alpha_{s}$ covaries with $\alpha_{s}+\alpha_{f}=10$.

**Figure 15:**
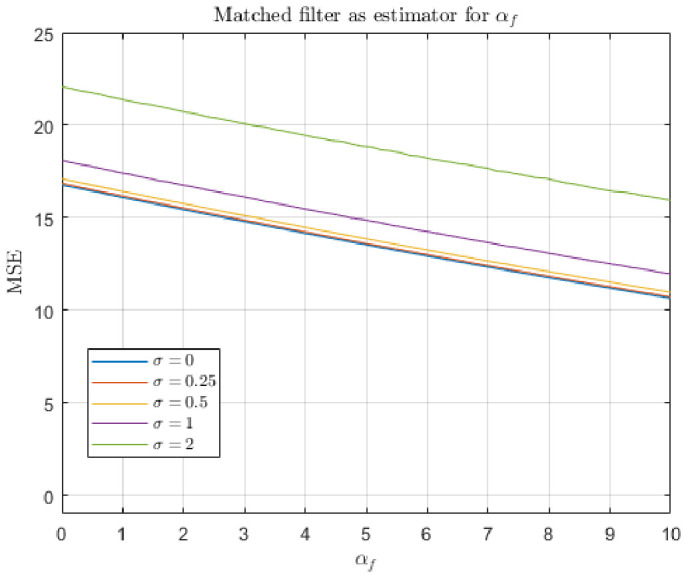
Mean-squared error of the matched filter response estimator for $\alpha_{f}$, collected while varying $\alpha_{f}$ and the additive noise magnitude σ. The skin radiance $\alpha_{s}$ covaries with $\alpha_{s}+\alpha_{f}=10$.

The results of the second simulation are shown in Figure 14 and Figure 15. In this scenario, the total radiant power of the input light is fixed. It appears that the non-negative least squares estimator is superior under these assumptions. Qualitatively, Figure 12 and Figure 14 appear very much the same, which implies this constraint does not affect the performance of the non-negative least squares very much.

In contrast, Figure 13 and Figure 15 are very different—when the constraint on total radiance is introduced, the matched filter response MSE decreases with $\alpha_{f}$, whereas it increased in the first simulation where total radiance increased with $\alpha_{f}$. Recall that in the second simulation, when $\alpha_{f}=0$, it must be the case that $\alpha_{s}=10$. Thus, the matched filter response’s tendency to confuse alternative light sources with Glo Germ inflates the output, leading to increased error magnitude.

### 4.3. Example of Hand Images

**Figure 16:**
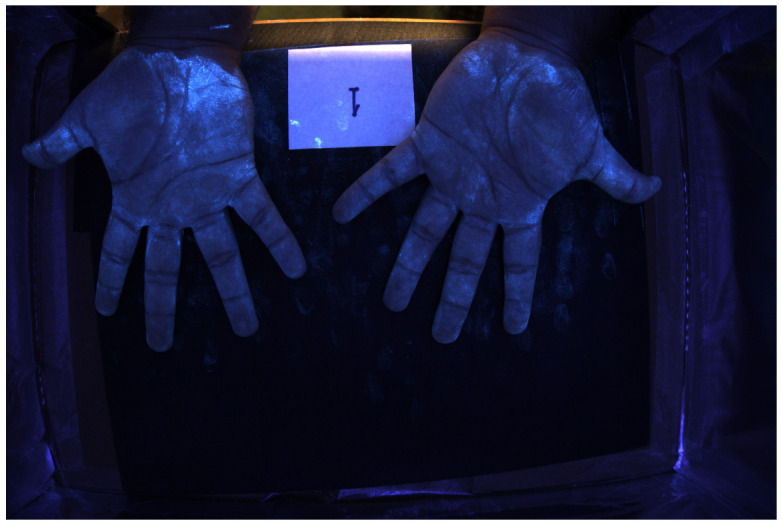
RGB image of hands covered in low-density Glo Germ.

**Figure 17:**
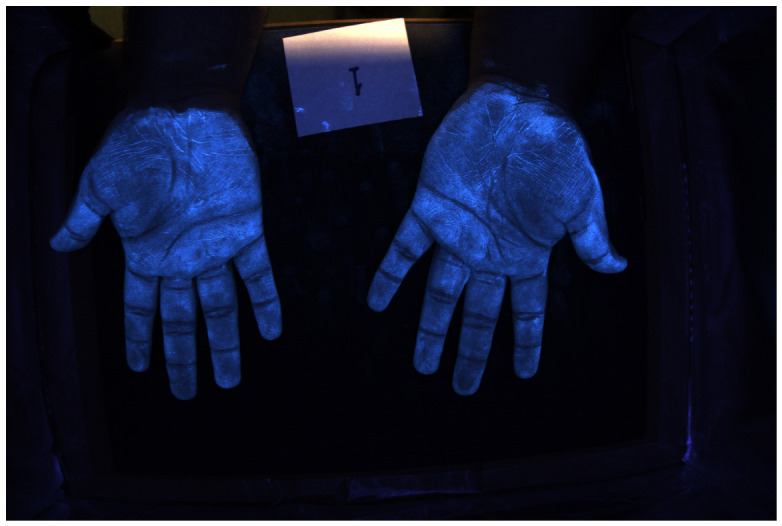
RGB image of hands covered in high-density Glo Germ.

Figure 16 and Figure 17 show color images from a camera capable of outputting a RAW file of hands covered in low and high-density Glo Germ (after and before washing). In theory, any camera that provides RAW files will work, as long as the user correctly transforms the RAW file into XYZ color space. In actual applications, these images should be cropped to remove the background but we include them uncropped here to illustrate how the method performs on wider features of the environment, such as the white card and portions of the background illuminated by the outside room.

**Figure 18:**
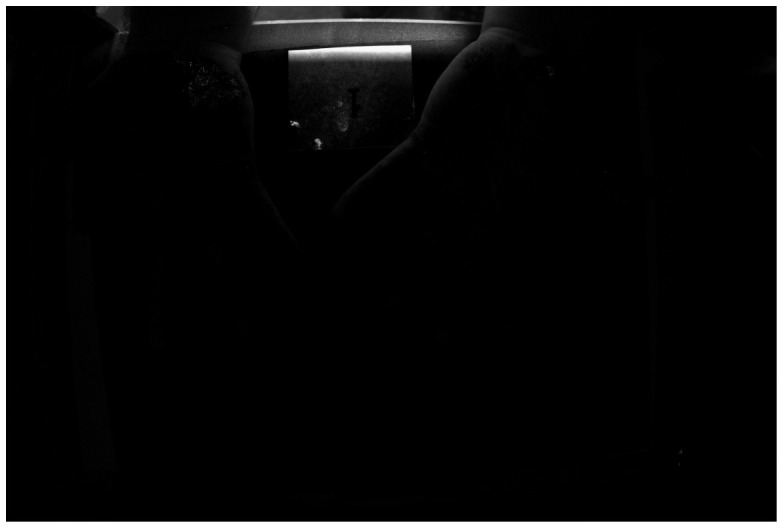
White light detected using the NNLS method decomposition applied to the color image in Figure 16.

**Figure 19:**
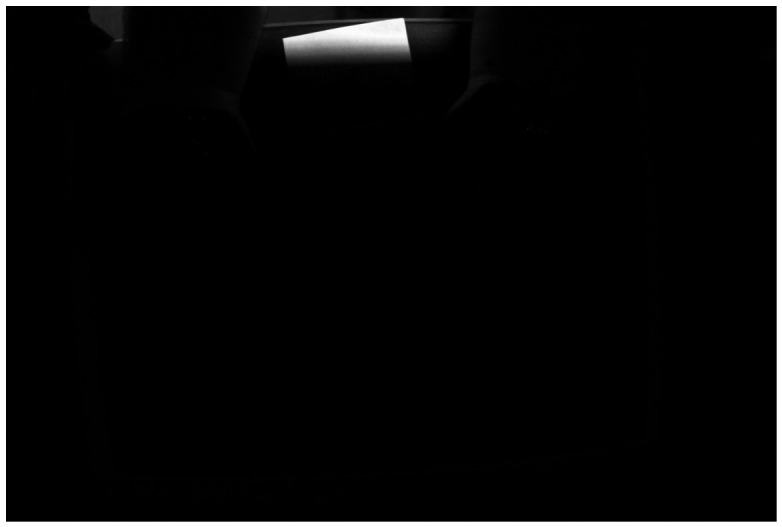
White light detected using the NNLS method decomposition applied to the color image in Figure 17.

For example, Figure 18 and Figure 19 show spatial maps of the non-negative coefficient $\beta_{w}\left(x,y\right)$, representing the contribution of white light to the measured color. These maps are especially sensitive to the contribution of non-UV illumination, as evidenced by the brightness of regions illuminated by the room. The room light reflecting off the white card, table, and skin surface is captured and its contribution is removed from the measured colors.

**Figure 20:**
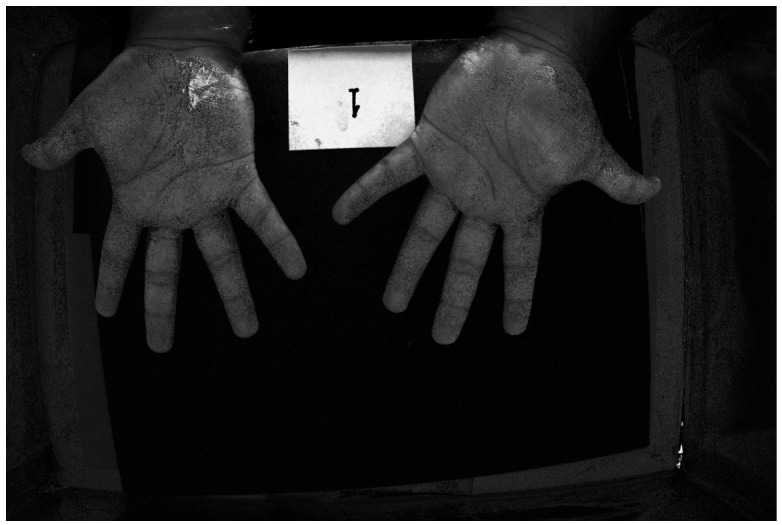
Skin radiance detected using the NNLS method decomposition applied to the color image in Figure 16.

**Figure 21:**
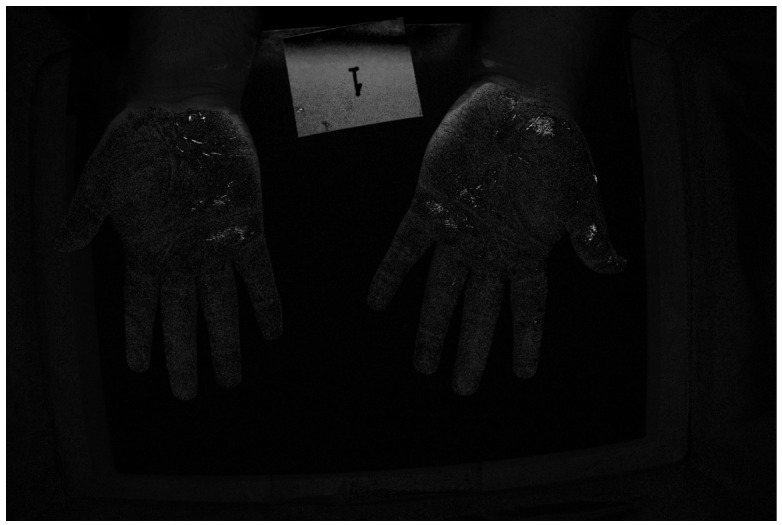
Skin radiance detected using the NNLS method decomposition applied to the color image in Figure 17.

Figure 20 and Figure 21 show spatial maps of the non-negative coefficient $\beta_{s}\left(x,y\right)$, representing the contribution of the UV-illuminant reflecting off the surface of the skin to the measured color. The fact that Figure 20 is brighter than Figure 21 is a testament to the fact that the skin is only visible at sufficiently low densities. Interestingly, the “skin detector” appears to confuse the white card for skin under the UV-illuminant. This is evidenced by the brightness gradient across the surface of the card as it transitions from room illuminant to UV.

**Figure 22:**
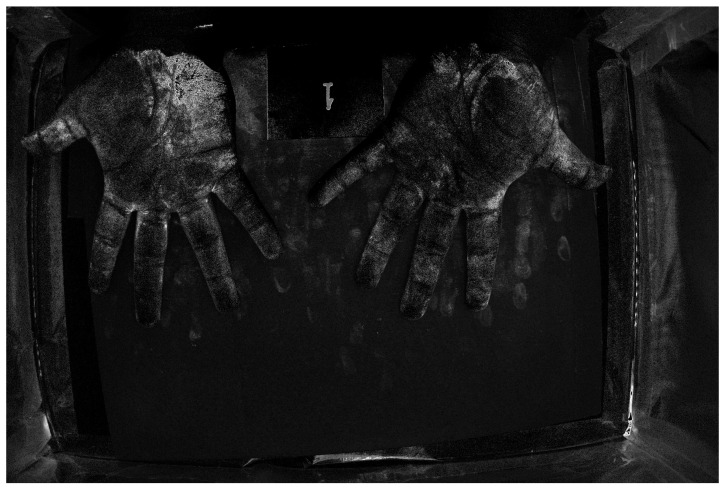
Fluorophore density detected using the NNLS method decomposition applied to the color image in Figure 16.

**Figure 23:**
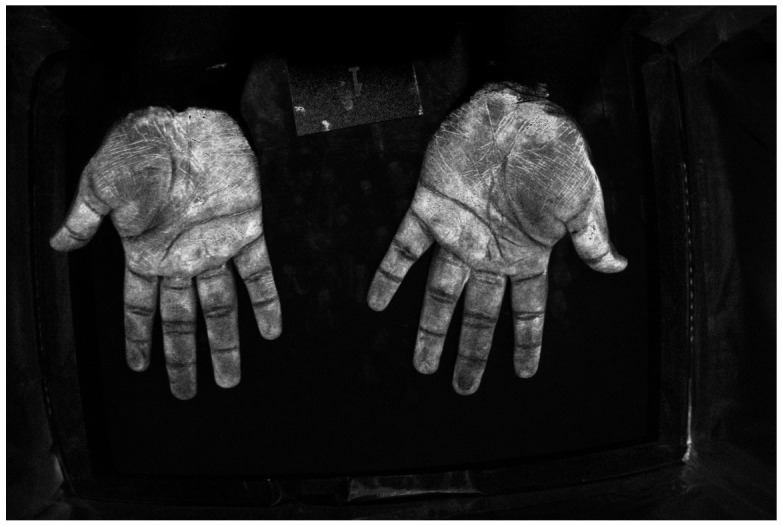
Fluorophore density detected using the NNLS method decomposition applied to the color image in Figure 17.

Finally, Figure 22 and Figure 23 show spatial maps of the non-negative coefficient $\beta_{f}\left(x,y\right)$, representing the contribution of fluorescent emissions to the measured color. These are very sparse maps, with many of the pixels taking a value of 0.

## 5. Discussion

### 5.1. Interpretation of the Linear Mixture Model

The central assumption of this work is that light measured by a camera pixel under ultraviolet illumination can be decomposed into additive contributions from (1) broadband white light, (2) surface reflectance of skin illuminated by the UV source, and (3) fluorescent emission from Glo Germ. Unlike classical histological stain analysis, where pigments combine subtractively and optical density models are required, fluorescence emission is additive (see Table 1 for a detailed comparison). Moreover, because Glo Germ is deposited on the surface rather than embedded volumetrically, the reflective component of the skin combines linearly with the emitted fluorescence over a wide operating range. This makes linear decomposition not only mathematically convenient but physically appropriate for the macroscopic regime considered here.

### 5.2. Practical Considerations

When these methods are used in applications, the images of hands should be cropped to exclude all surfaces except human skin. This can be done manually with tools, or automatically with segmentation software designed for human anatomy [22]. The test photos provided in the Supplementary Materials include masks to remove the background.

The experimental results also provide clear guidance for selecting camera parameters in practice. Across both the empirical response curves (Figure 8 and Figure 10) and the simulation results (Figure 12, Figure 13, Figure 14 and Figure 15), estimator performance is improved in regimes where measured intensities remain low and sensor saturation is avoided. In this regime, the linear mixture assumption remains valid, the matched filter response retains discriminability, and the NNLS estimator avoids misclassification of fluorescence as broadband illumination. Consequently, optimal imaging conditions favor minimizing total incident intensity at the sensor. In practice, this corresponds to using the lowest feasible ISO setting and the shortest exposure duration that still provides sufficient signal above the noise floor. Operating in this low-intensity regime reduces both saturation-induced nonlinearities and estimator bias, yielding lower mean-squared error and more reliable paired comparisons. A notable exception arises in scenarios where total radiance is effectively constrained and only chromatic composition varies (i.e., $\alpha_{s}+\alpha_{f}$ is fixed). Under these conditions, the matched filter response benefits from increased fluorescence contribution, as its mean-squared error decreases with $\alpha_{f}$ due to reduced confounding from background components. Thus, while low-intensity operation is generally optimal for the NNLS-based decomposition and for maintaining physical interpretability, higher relative fluorescence levels may be advantageous when using matched filter responses in chromatically constrained settings.

### 5.3. Relationship to Prior Work

The mathematical structure of the proposed estimator further clarifies its relationship to matched filtering, and particularly orthogonal subspace projection (OSP) [23]. In classical matched filtering, the detection statistic is obtained by projecting the observation vector L(I) onto the known target spectrum $L(\hat{f})$. In hyperspectral target detection, this idea is extended by first removing the contribution of known background spectra (in our case, $L(\hat{s})$ and L(w)) before evaluating the target response. The OSP detector accomplishes this by projecting the observation onto the orthogonal complement of the background subspace, and then correlating the residual with the target signature. The present method performs an equivalent operation through linear mixture estimation. By modeling each pixel as a linear combination of white light, skin reflectance, and fluorescence, the regression implicitly removes the components of the measured color that lie in the subspace spanned by the background spectra. The estimated fluorescence coefficient therefore represents the magnitude of the fluorescence spectrum after accounting for these competing sources of light. In this sense, the coefficient $\beta_{f}$ plays the role of a background-suppressed matched-filter response, while the non-negative least-squares constraint ensures that the resulting decomposition remains physically plausible. Thus, the proposed observer can be interpreted as a constrained implementation of a background-orthogonalized matched filter operating in tristimulus color space.

Recent work in image restoration has demonstrated the effectiveness of combining physics-based forward models with structured priors to address ill-posed inverse problems. For example, ref. [24] introduce the Region Line Prior (RLP) for image dehazing, which exploits an empirically observed quasi-linear relationship between regional brightness in hazy and haze-free images, reducing the dehazing problem to the estimation of a small number of parameters via global optimization. Similarly, ref. [25] propose the Inverted Haze Density Correction Prior (IHDCP), which augments the atmospheric scattering model with a pixel-wise nonlinear prior linking transmission to haze density through a gamma correction function. While these approaches share with the present work the general strategy of embedding prior structure within a forward image formation model to make inversion tractable, they differ in both domain and formulation. Specifically, IDRLP and IHDCP operate in the spatial domain and rely on empirically derived relationships between image statistics and latent variables, with the primary goal of visual image restoration. In contrast, the method proposed here operates in colorimetric space and derives its constraints from measured spectral power distributions and radiometric principles, enabling physically interpretable decomposition of fluorescence and reflectance components for quantitative analysis rather than perceptual enhancement.

Recent work in computer vision has emphasized the importance of robust feature representations for separating relevant signals from complex backgrounds [22]. While such approaches typically rely on deep learning and large datasets, the present work addresses a related problem through a physics-based model observer that separates fluorescence from background reflectance using measured spectral information. Finally, the approach is structurally analogous to color-deconvolution methods used in digital histology, where stain densities are estimated from RGB images using known chromatic signatures. The key difference is that histological stains combine multiplicatively through absorption and are therefore modeled in optical density space, whereas fluorescence emission adds radiance to reflected light and can be modeled directly as a linear mixture in intensity space. Viewed in this way, the proposed method represents a low-dimensional spectral unmixing framework that integrates ideas from signal detection theory, hyperspectral target detection, and color deconvolution into a single physically interpretable model observer.

Importantly, while many of these methods are typically described in high-dimensional spectral spaces, the present results demonstrate that the same principles apply in the low-dimensional setting of camera tristimulus values, provided the target spectrum is measured and mapped correctly into camera space.

### 5.4. Comparison of Matched Filter Response and Non-Negative Least Squares Estimation

The matched filter response and the non-negative least squares (NNLS) estimator represent two closely related but qualitatively different approaches to estimating the fluorescent component of the measured signal. While both are derived from the same underlying linear model (Equations (3) and (11)), their behavior differs substantially due to the presence or absence of source separation and constraints.

First, the two estimators differ in their behavior at high intensities. As shown in the response curves (Figure 8 and Figure 10), the matched filter response does not catastrophically fail under high exposure conditions. Instead, it exhibits a saturation effect, where the response asymptotically approaches a maximum value and loses the ability to discriminate between higher concentrations of Glo Germ. In contrast, the NNLS estimator can fail more abruptly when the camera sensor becomes saturated. Under these conditions, the observed color shifts toward the white-light direction, causing the decomposition in Equation (11) to misclassify fluorescence as broadband illumination. Thus, while the matched filter is limited by saturation, the NNLS estimator is sensitive to violations of the linear radiometric assumption.

Second, the NNLS estimator explicitly separates sources of light, whereas the matched filter response does not. The matched filter projects the observed color onto the fluorescence direction $L(\hat{f})$, but any component of the signal that is correlated with this direction—such as UV-illuminated skin reflectance or residual white light—will contribute to the response. As a result, the matched filter confounds fluorescence with other sources of light. In contrast, the NNLS estimator partitions the measured signal into components aligned with L(w), $L(\hat{s})$, and $L(\hat{f})$, effectively removing the contributions of background spectra before estimating fluorescence. This difference is evident in both the simulated results and the real-image examples, where the NNLS-derived fluorescence maps suppress background structure that remains visible in single-channel proxies.

Third, the statistical behavior of the estimation errors differs markedly between the two methods. In the simulations described in Section 4.2, the NNLS estimator produces bimodal error histograms. One mode occurs when the estimator assigns $\beta_{f}=0$, corresponding to cases where the fluorescence signal is weak relative to noise or background components. The second mode occurs when the estimator correctly recovers $\beta_{f}\approx \alpha_{f}$, with errors centered on zero. This reflects the constrained nature of the estimator, which either attributes no fluorescence or assigns a physically meaningful positive value. In contrast, the matched filter response produces approximately normal error distributions, consistent with its derivation under Gaussian noise assumptions. However, these distributions are centered at a positive mean, indicating a systematic overestimation of fluorescence due to the inclusion of background components that are correlated with $L(\hat{f})$.

Fourth, images of fluorophore density obtained from NNLS tend to have higher image contrast than images formed from the matched filter response. For example, in the low (high)-density photo shown in Figure 16 and Figure 17, the NNLS image has a root-mean squared contrast of 1.184 (1.685), whereas the matched filter response image has a RMSC of 0.975 (1.312). In other words, the NNLS image has approximately 21.4% (28.4%) more image contrast than the matched filter response image. This is due to two things: First, the matched filter response confuses alternative light sources for Glo Germ fluorescence. Second, the bimodal error histogram of the NNLS estimator is caused by zeros being assigned to many pixels, often next to a neighbor with high Glo Germ density. It is important to note that higher contrast does not necessarily guarantee a higher quality image—as almost any image can be transformed into one with higher contrast by randomly setting some pixels to zero. Obviously in this case, the destruction of information cannot lead to a higher quality photo.

Finally, the NNLS estimator appears more robust to changes in the underlying assumptions about the signal. This is evident in the mean-squared error (MSE) simulations (Figure 12, Figure 13, Figure 14 and Figure 15), where modifying the constraint on total radiance (i.e., fixing $\alpha_{s}$ versus fixing $\alpha_{s}+\alpha_{f}$) does not qualitatively change the behavior of the NNLS estimator. In both cases, the MSE curves exhibit similar shapes and asymptotic behavior. In contrast, the matched filter response is highly sensitive to these assumptions. When total radiance is allowed to increase with $\alpha_{f}$, the MSE increases with fluorescence intensity; when total radiance is fixed, the MSE decreases with $\alpha_{f}$. This sensitivity arises because the matched filter does not explicitly model competing sources of light, and therefore, its output depends strongly on how those sources covary with fluorescence.

Taken together, these results highlight a fundamental trade-off between the two approaches. The matched filter provides a simple, stable projection that behaves predictably under noise and saturation but lacks the ability to disentangle competing sources of light. The NNLS estimator, by contrast, achieves source separation and improved interpretability at the cost of increased sensitivity to violations of the linear model, particularly under sensor saturation. For the paired-comparison setting considered in this work, where images are acquired under controlled conditions and exposure can be managed, the advantages of source separation outweigh these limitations, making the NNLS estimator a more suitable choice for quantifying changes in fluorescence.

We invite readers to use the MATLAB function included in the Supplementary Materials (CompareMeasures.m) to compare the estimators generated from whatever images the readers provide. Simply use the function raw2xyz.m to convert a RAW file into XYZ coordinates, then provide the XYZ image as the input to CompareMeasures.m. The function yields two outputs. The first is the estimated glow using non-negative least squares, and the second is the matched filter response.

### 5.5. Why Not Just Use the Blue Channel?

**Figure 24:**
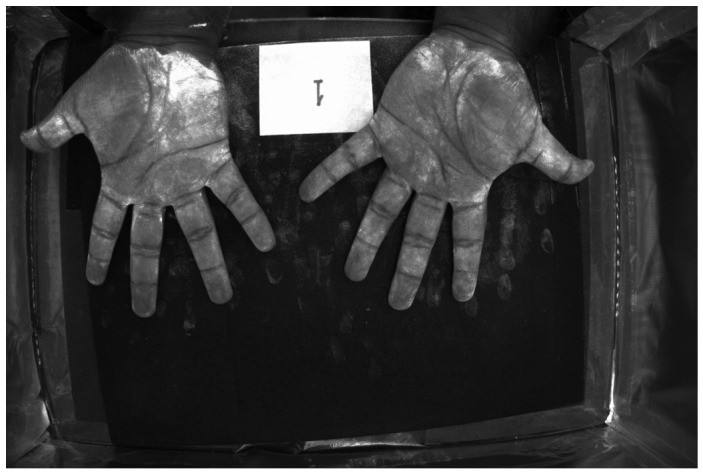
Fluorophore density detected using the blue channel from Figure 16.

**Figure 25:**
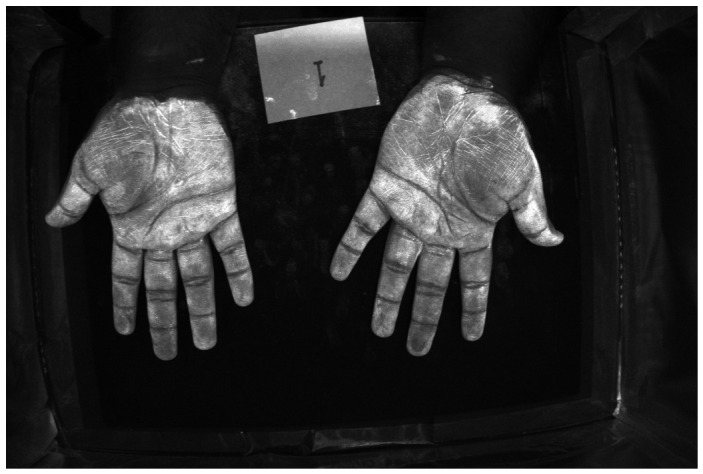
Fluorophore density detected using the blue channel from Figure 17.

Figure 24 and Figure 25 show what happens when we use the camera’s blue channel response as a proxy for Glo Germ density. The proxy does an excellent job of detecting the blue light contribution of Glo Germ. However, like the mathced filter response, it also conflates the contribution of Glo Germ with the contributions of white light and skin reflectance. This is obvious by the visibility of the white card and wrists in the photo. Consider the fact that under the presence of UV illumination, most of the photons available for reflecting off surfaces will be limited to wavelengths less than 450 nm—thus, most of the light available for the camera sensors will be “blue.” This is particularly noticeable in the low-density photos (Figure 22 and Figure 24). As a result, the blue channel images have lower contrast than the NNLS images in Figure 22 or Figure 23. Measured as root-mean squared contrast, the images in Figure 22 and Figure 23 have, respectively, 22% and 28% more image contrast than their counterparts in Figure 24 and Figure 25. The blue channel image is nearly identical to the matched filter response image, which makes sense because they are merely linear transformations of each other. In summary, the blue channel is at best a poor man’s approximation of the matched filter response, but it can be used in much the same way.

### 5.6. Limitations and Future Directions

Several limitations should be noted. First, the observer assumes that camera data are linear with respect to incident radiance. Violations due to saturation or nonlinear response curves will degrade performance. Second, XYZ space is used as a camera-agnostic proxy; operating directly in linear sensor space with explicitly measured spectral sensitivities would further improve optimality. We decided to use XYZ coordinates because they are universal. Provided a reader is able to correctly transform their camera sensor readings into XYZ coordinates, they can use the projection basis reported here (Equation (8)) to reproduce the decomposition. Third, the model does not explicitly account for inelastic scattering or secondary fluorescence effects, which may contribute modestly to the measured emission spectrum.

Future extensions could include multi-fluorophore decomposition, or variable modeling of spatially varying backgrounds, instead of skin reflectance. More broadly, the framework generalizes to any application in which a characteristic emission or reflectance spectrum can be measured independently and projected into camera space.

## 6. Conclusions

We presented a spectrally informed, model-observer framework for quantifying Glo-Germ fluorescence in images acquired under controlled conditions, with a specific focus on paired comparisons before and after hand washing. By combining measured emission spectra with a linear mixture model in CIE XYZ space, the method separates fluorescence from background reflectance using non-negative least squares and produces a scalar metric proportional to total fluorescent material.

Compared to simple projection-based approaches, the NNLS estimator provides improved interpretability and background suppression, enabling more reliable quantification in controlled experiments. Both simulations and empirical results indicate that operating in a low-intensity regime is critical for maintaining linearity and minimizing error.

Rather than addressing general fluorescence detection, this work establishes a reproducible and physically grounded approach for quantitative comparison of contamination levels under fixed imaging conditions.

## Figures and Tables

**Table 1:** Comparison between additive fluorescence modeling and subtractive staining (color deconvolution) approaches.

Aspect	Additive Fluorescence Modeling	Subtractive Staining (Color Deconvolution)
Physical process	Fluorophores emit light under excitation (radiance is added)	Stains absorb light passing through tissue (radiance is attenuated)
Image formation model	Linear combination in intensity space: $I=\sum_{i}\beta_{i}L_{i}$	Multiplicative attenuation: $I=I_{0}e^{-\sum_{i}a_{i}k_{i}}$
Linearization	Already linear in radiance; no transform required	Log transform required: $OD=-\log(I/I_{0})$
Domain of analysis	Linear color space (e.g., XYZ or linear RGB)	Optical density (OD) space
Basis vectors	Emission/reflectance spectra projected into color space	Stain absorbance spectra
Unknowns	Source contributions (e.g., fluorescence, reflectance, illumination)	Stain concentrations
Estimation method	Linear unmixing (e.g., non-negative least squares)	Linear unmixing in OD space (matrix inversion or regression)
Effect of increasing concentration	Increases total intensity (additive)	Decreases transmitted intensity (subtractive)
Failure mode	Sensor saturation leading to loss of linearity and source confusion	Low signal leading to instability after log transform
Interpretability	Coefficients correspond to emitted radiance contributions	Coefficients correspond to absorbing material density
Typical applications	Fluorescence imaging, spectral detection, remote sensing	Histology, pathology (e.g., stain separation)

**Figure A1:**
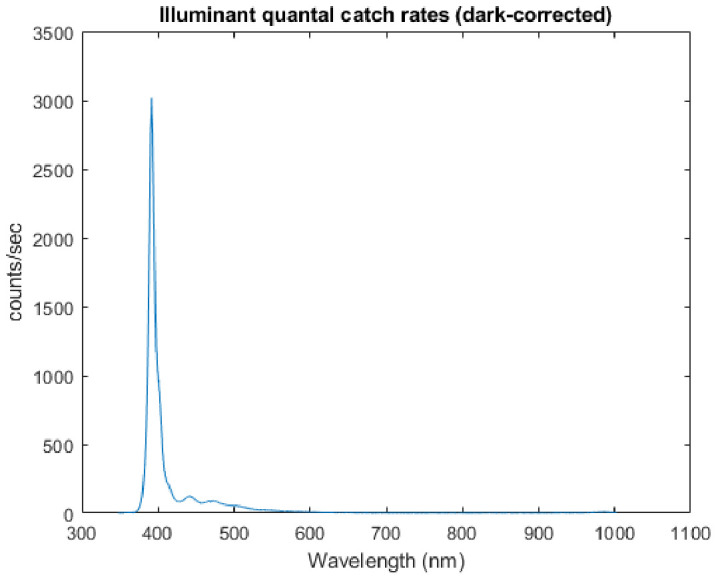
Quantal emission spectrum of the illuminant, following dark-correction only. The units are the raw instrumental counts per second of integration time.

**Figure A2:**
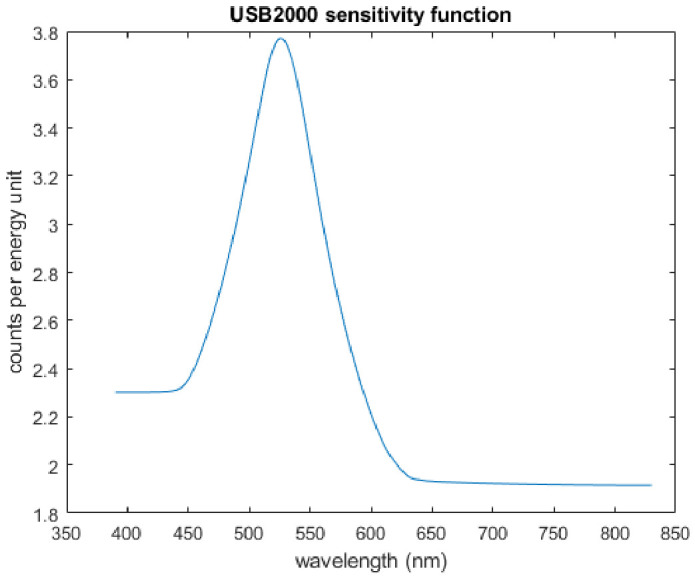
Sensitivity function of the USB2000 estimated from the Dell UP2720Q LED monitor with a white point approximately 6500 K. Performing the irradiance correction with this function imparts an energy-based metric on the spectra, making them suitable for colorimetric computations.

## Data Availability

Data are available upon reasonable request.

## Supplementary Materials

The following supporting information can be downloaded at: https://drive.google.com/file/d/1WYcaS2iHyJ5bOY4U2dKp-g8CDaazGim7/view (accessed on 7 April 2026). “Random photos” is a folder containing a photo of a nitrile glove and a white t-shirt in order to illustrate how the method works when applied to random objects. “Raw spectral data” is a folder containing the raw spectral photometric data recorded by our USB2000. “Response curve photos” contains photographs we used to obtain the response curves as a function of concentration. “Test photos” contains photos of hands covered in GloGerm for testing the method. The folder also includes TIFF and XMP files containing a mask for the hands to remove the background. The easiest way to run the code is to open the MATLAB function ViewTestImages.m and change the variable imgdir to match the location of the test photos folder.

## Abbreviations

The following abbreviations are used in this manuscript:

SPDF	Spectral power density function
NNLS	Non-negative least squares
MSE	Mean squared error

## Appendix A. Spectral Photometer Calibrations

### Appendix A.1. Apparatus

#### Appendix A.1.1. Enclosure and Stray-Light Control

The entire apparatus was contained in a large cardboard box with the interior spray painted matte-black. This enclosure served three purposes:

1. reduction of ambient light contamination,
2. suppression of internal reflections and veiling glare,
3. improved measurement repeatability by stabilizing geometry and illumination.

#### Appendix A.1.2. Target Placement

A blob of Glo Germ, approximately 3 cm in diameter, was positioned in the center of one wall of the box. The blob served as the fluorescence target.

#### Appendix A.1.3. Spectrometer Geometry (USB2000 + Collimating Lens)

The collimating lens of the USB2000 (Manufactured by Ocean Optics, Orlando, FL, USA) was positioned at the same height as the blob and 36 cm away, angled perpendicular to the surface of the blob. The fiber optic cable carried the collimated light to the USB2000, located outside the box.

Because the lens has a field of view (FOV) less than 2.5°, the measurement sampled a small solid angle centered on the blob. This geometry helps isolate the blob from surrounding surfaces.

#### Appendix A.1.4. UV Illuminant

The illuminant was a 24 watt LED blacklight with a peak wavelength between 395 and 405 nm, produced by YGS-Tech (Hialeah, FL, USA). The 165 LED lights were arrayed along 11 strips and affixed to the edges of the box opposite the blob. This configuration maximized UV exposure of the target while allowing the spectrometer to view the target through the collimating lens and fiber optic cable.

### Appendix A.2. Measurement Procedure

#### Appendix A.2.1. Overview

Two spectra are typically useful:

1. the illuminant spectrum (UV source spectral power distribution when viewed directly by the collimating lens),
2. the fluorescent emission spectrum (radiance from the Glo Germ blob under UV excitation).

#### Appendix A.2.2. Acquisition Steps

1. **Warm-up:** Allow the UV lights to reach stable output.
2. **Dark measurement:** With the collimating lens covered, record a dark spectrum D(λ) using the same integration times and settings as later measurements.
3. **Illuminant measurement:** Configure the spectrometer to measure the UV source spectrum in the same enclosure. Record $I_{\mathrm{meas}}\left(\lambda \right)$ and compute the dark-corrected illuminant:
  (A1) $$I\left(\lambda \right)=I_{\mathrm{meas}}\left(\lambda \right)-D\left(\lambda \right).$$
4. **Fluorescence measurement:** Position the spectrometer FOV on the blob (as described above). With UV lights on, record $F_{\mathrm{meas}}\left(\lambda \right)$ and compute dark-corrected signal:
  (A2) $$F\left(\lambda \right)=F_{\mathrm{meas}}\left(\lambda \right)-D\left(\lambda \right).$$

#### Appendix A.2.3. Instrument Considerations

##### Integration Time and Saturation

Integration times are chosen to avoid saturation near the signal peak while maximizing signal-to-noise ratio (SNR). For the illuminant, our integration time was 1200 ms. For the target, integration time was 1500 ms.

##### Repeated Measurements

Raw measurements are repeated 100 times, so downstream computations are based on averages with a large sample size.

##### Wavelength Sampling

The USB2000 provides spectra sampled at discrete, unevenly-spaced wavelengths. Downstream computations (tristimulus integrals) are corrected for the sampling interval Δλ by integration of the interpolant over 1 nanometer intervals centered on wavelengths 390 to 830. This allows for the calculation of tristimulus values using standard matrix multiplication.

##### Absolute Irradiance Correction

In practice, the USB2000 measures spectral intensity in instrument units; the analysis can be performed either in relative units (normalized spectra) or in radiometrically calibrated units if a calibration standard is available. The USB2000 has a sensitivity function that varies systematically across wavelengths. This imparts a bias in raw measurements that must be corrected for by estimating the inverse sensitivity function. This estimation procedure involves measuring the spectrum of a broadband/white light source with known colorimetric coordinates. In the present case, that light was the D65 white point of a Dell UP2720Q LED monitor.

If S(λ) is the sensitivity of the USB2000 to photons with wavelength λ, then the final corrected fluorescent emission spectrum is:

(A3) $$\hat{F}\left(\lambda \right)=\frac{F(\lambda )}{S(\lambda )}.$$
